# Irregular Bone Defect Repair Using Tissue-Engineered Periosteum in a Rabbit Model

**DOI:** 10.1007/s13770-020-00282-4

**Published:** 2020-09-10

**Authors:** Lin Zhao, Junli Zhao, Jia-Jia Yu, Cangyu Zhang

**Affiliations:** 1 Orthopaedic Department of Guangming Traditional Chinese Medicine Hospital of Pudong New Area, Shanghai, 201399 People’s Republic of China; 2 Department of Nephrology, Shanghai University of Medicine & Health Sciences affiliated Zhoupu Hospital, Pudong New District, Shanghai, 201318 People’s Republic of China; 3 Department of the Joint Surgery, Yuncheng Central Hospital, Hongqi West Street 173, Yanhu District, Yuncheng City, 044000 Shanxi Province People’s Republic of China; 4 Orthopaedic Department of the 2nd Hospital of Lanzhou University, 80 Cui Ying Men, Cheng Guan District, Lanzhou City, 730030 People’s Republic of China

**Keywords:** Tissue engineering, Periosteum, Irregular bone, Bone defect

## Abstract

**Background:**

In previous studies, we succeeded in repairing a long bone defect with tissue-engineered periosteum (TEP), fabricated by incorporating rabbit mesenchymal stem cells with small intestinal submucosa. In this study, we investigated the feasibility of allogeneic irregular bone defect repair using TEP.

**Methods:**

We performed a subtotal resection of the scapula in 36 rabbits to establish a large irregular bone defect model. The rabbits were then randomly divided into three groups (n = 12 per group) and the defects were treated with TEP (Group 1), allogeneic deproteinized bone (DPB) (Group 2) or a hybrid of TEP and DPB (Group 3). At 4, 8, and 12 weeks after surgery, the rabbits were sacrificed, and the implants were harvested. X-ray radiographic and histological examinations were performed to detect bone healing. Ink-formaldehyde perfusion was introduced to qualitatively analyze vascularization in TEP engineered new bone.

**Results:**

The repair of scapular defects was diverse in all groups, shown by radiographic and histological tests. The radiographic scores in Group 1 and Group 3 were significantly higher than Group 2 at 8 and 12 weeks (*p* < 0.05). Histological scores further proved that Group 1 had significantly greater new bone formation compared to Group 3 (*p* < 0.05), while Group 2 had the lowest osteogenesis at all time-points (*p* < 0.001). Ink-formaldehyde perfusion revealed aboundant microvessels in TEP engineered new bone.

**Conclusion:**

We conclude that TEP is promising for the repair of large irregular bone defects. As a 3D scaffold, DPB could provide mechanical support and a shaping guide when combined with TEP. TEP engineered new bone has aboundant microvessels.

## Introduction

Massive bone defects remain a challenge for orthopedic surgeons [1–3]. Bone tissue engineering (BTE) is a promising approach for bone defect repair. The classical BTE approach is to select a biomaterial scaffold that provides structural support for 3D bone tissue formation [4, 5]. However this results in limited bone tissue regeneration, mainly due to insufficient delivery of nutrients and oxygen and metabolic waste removal within the 3D scaffolds [6]. Seeding cells onto the outer surfaces of 3D scaffolds may allow cells to access sufficient nutrients, but cells located inside the scaffolds would be likely to undergo necrosis, which hinders bone regeneration [7, 8]. In addition, in the absence of a capillary network within the 3D implants, engineered tissues can only have a maximum thickness of 150–200 mm; dimensions larger than this threshold may result in a lack of oxygen inside the biomaterials [9, 10]. The existence of so many formidable conceptual and technical challenges impede clinical translation of experimental successes into clinical practice [5]. In the clinic, bone defects occur in any size and shape as a result of surgical treatment of tumors and other bone diseases. Traditional treatment is not likely to restore their original size and shape. Therefore, intensive efforts should be made to seek an alternative approach.

The periosteum plays an indispensable role in both bone formation and bone defect healing via an endogenous repair approach [11–13]. Some papers have illustrated an approach using mesenchymal stem cell (MSC) sheets or periosteum for bone healing [14–16]. Fabrication of a biomimetic periosteum substitute that could fit any size and shape of bone defect would be a promising approach to bone defect repair [14].

Based on tissue-engineering principles, in previous studies, we developed a flexible cellular construct that serves as an osteogenic and angiogenic ‘‘periosteum,” a kind of homemade tissue-engineered periosteum (TEP), which was fabricated by incorporating osteogenically-induced rabbit MSCs with a scaffold of small intestinal submucosa (SIS). It has successfully been used to reconstruct long bone critical-size defects (CSDs) in our previous studies [17, 18]. In this study, we hypothesize that TEP may repair a large irregular bone defect in a rabbit model.

## Materials and methods

### Animals

Forty-six New Zealand white rabbits (NZWRs), consisting of 36 adults (2 months of age, approximately 2.0 kg) and 10 neonatal rabbits (2 weeks, approximately 0.40 kg), were provided by the Animal Experiment Center of the Lanzhou Institute of Biological Products (Lanzhou, Gansu province, China). All of the experimental procedures involving animals were conducted in accordance with the Institutional Animal Care Guidelines of Lanzhou University, China, and were approved by the Institutional Animal Care and Use Committee of Lanzhou University, Gansu Province, China (IACUC no. 2016016).

### Cell culture

Cell culture were performed as in previous studies [17, 18]. Briefly, bone marrow (5 mL) was aspirated from the ventral ilium of neonatal NZWRs (2 weeks, approximately 0.40 kg). MSCs isolated from bone marrow were collected and then plated into a plastic culture flask with Dulbecco’s Modified Eagle Medium (DMEM, Invitrogen, Carlsbad, CA, USA) containing 10% fetal bovine serum (FBS, Sijiqing, Hangzhou, China) and incubated at 37 °C with 5% CO_2_. The primary passage MSCs were observed under a microscope. When MSCs reached 80–90% confluence, they were detached with 0.25% trypsin (Gibco, Carlsbad, CA, USA), transferred to new culture flasks at a density of 2 × 10^6^ L^−1^ and subcultured two times upon reaching 90% confluence.

### Osteogenic induction

Passage three MSCs were used for osteogenic differentiation in standard DMEM supplemented with 50 mg L^−1^ ascorbic acid (Sigma-Aldrich, St. Louis, MO, USA), 10 mmol L^−1^ sodium β-glycerophosphate (Sigma-Aldrich) and 10^−8^ mol L^−1^ dexamethasone (Sigma) at 37 °C in a humidified 5% CO_2_ incubator for 3 weeks.

Osteogenic differentiation of MSCs was idenified by modified Gomori staining (Yuanmu Biotech, Shanghai, China) and Alizarin Red (Sigma-Aldrich) staining with conventional methods.

After characterization, the osteogenically-induced MSCs were collected as seeding cells.

### SIS preparation and TEP fabrication

Porcine small intestine, collected from healthy pigs (Lanzhou slaughter factory) within 4 h after slaughter, was cut into lengths of approximately 10 cm each. Submucosa was obtained by mechanical removal of the tunica serosa and muscularis. Then, the remaining submucosal layer was treated with a series of chemical decellularization steps, given detergent treatment, lyophilized, and sterilized [19]. Finally, all the samples were freeze-dried at − 70 °C in a lyophilizer, sealed into hermetic packages, and then sterilized using Co-60 gamma irradiation (25–35 kGy).

The SIS was clipped into squares (5 × 5 cm) and sterilized again under ultraviolet light for 2 h, then soaked in DMEM containing 20% FBS for 1 day before cell seeding. The suspension of osteoinduced MSCs (2.0 × 10^9^ L^−1^) was slowly dripped onto the SIS squares in culture dishes and incubated for 3 h at 37 °C. Then an appropriate volume of DMEM with 10% FBS was added to each composite, and incubated for a further 7 days.

### Scanning electron microscopy (SEM)

Some of the TEP, which was cultured for 15 days, was collected for inspection by SEM (JSM-680LA, Jeol Ltd., Tokyo, Japan). Briefly, samples were fixed in 2.5% glutaraldehyde for 7 days at room temperature, followed by washing thrice in PBS for 15 min each. Next, the specimens were subjected to critical point drying, then gold sputter coating before viewing by SEM.

### Preparation of deproteinized bone (DPB)

Fresh scapular bodies harvested by the surgical procedure of subtotal scapulectomy from NZWRs, were deproteinized in whole bone blocks after soft tissue removal. A dense array of vertical holes (each 1.5 mm in diameter) was drilled into each block to perform well deproteinization. The bone blocks were treated sequentially with H_2_O_2_, NaN_3_, NaOH, protease, methanol/chloroform mixture, ether, ethane diamine and absolute alcohol to produce DPB [20]. The samples were dried at 50 °C in a dry oven, sealed into hermetic packages, and then sterilized using Co-60 gamma irradiation (25–35 kGy).

### Animal surgery of irregular defect creation and treatment

All animal procedures were carried out in strict accordance with the regulations governing medical animal experiments. Thirty-six NZWRs (2 months, approximately 2.0 kg) were anesthetized intraperitoneally with an injection of 3% pentobarbital solution (40 mg/kg body weight). The unilateral shoulder of the rabbits was skinned and disinfected. Then, the scapular body with periosteum attached was exposed by bluntly separating the muscles, and resected off except for the glenoid and part of the scapular neck, the angulus superior and inferior to establish a subtotal scapulectomy model. This model aimed to preserve glenohumeral joint function for animal movement and prepare triangle anchoring points for implant attachment. Bone blocks from resection were removed together with the attached periosteum.

After creation of a unilateral segmentally-irregular bone defect in the shoulder blade of each animal, the 36 rabbits were divided randomly into three groups (n = 12 per group) and treated with TEP (Group 1), allogeneic DPB (Group 2), or TEP–DPB hybrid (Group 3).

**Fig. 1:**
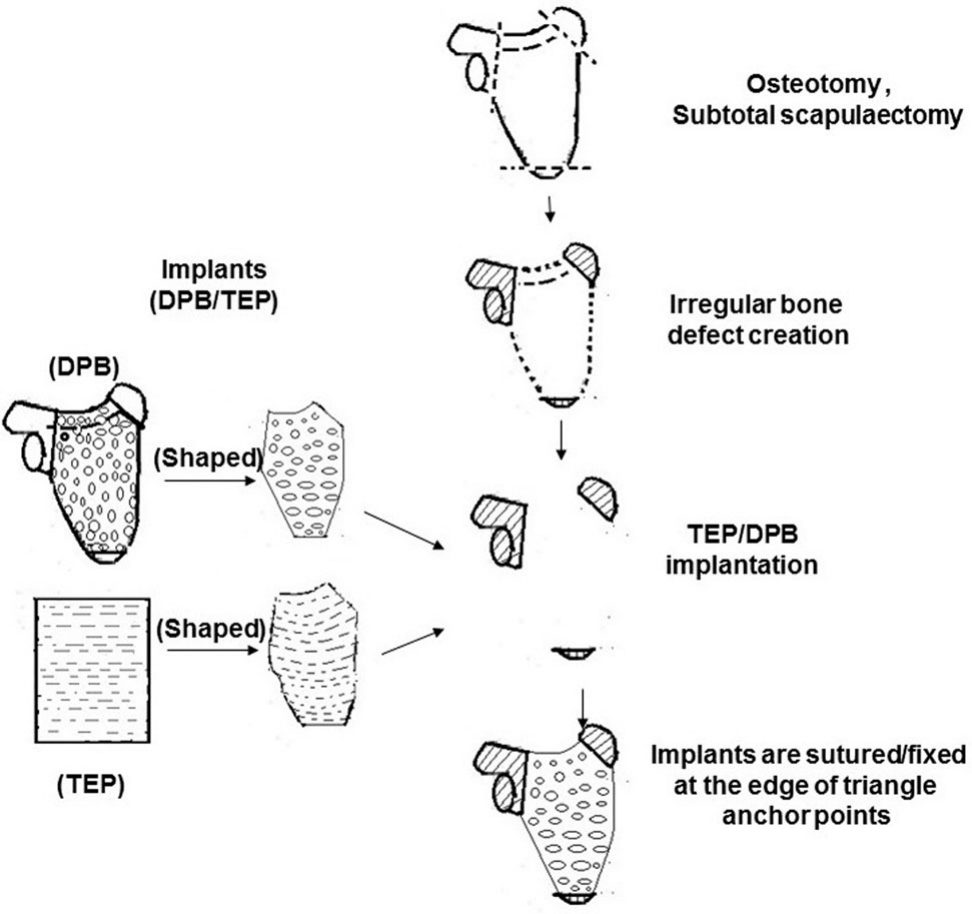
Schematic drawing of the surgical technique. Creation of a segmentally irregular bone defect model and repair using shaped implants

In Group 1, TEP was spread over the defect area and the margins were trimmed to fit the size and shape of the bone defect, then it was sutured with 7–0 microsurgical suture to the three prepared bony anchor points which were pre-drilled with several holes to allow a K-wire suture to pass through. In Group 2, an allogeneic DPB block was fixed with steel wire (0.5 mm diameter) to the three bony anchoring points. In Group 3, TEP enveloped the DPB. Briefly, TEP was wrapped around the surface of the DPB and fixed with 7–0 microsurgical sutures through vertical holes in the DPB using a puerperal suture technique. Then the hybrid implants (TEP-covered DPB) were fixed with steel wire (0.5 mm diameter) to the three bony anchor points. Once the implants were fixed tightly to the bony anchor points using tension sutures or K-wires, the incision was closed layer by layer with a 1–0 nylon suture. The rabbits received 400,000 units penicillin preoperatively and at the first/next postoperative day. The forelimbs and shoulders of each animal were immobilized on the surgical side with a plaster cast for 4 weeks. The surgical procedure described is depicted in a schematic drawing, shown in Fig. 1.

At 4, 8, and 12 weeks after surgery, 3 ~ 4 rabbits (3 in group 1 at 12 weeks) in each group were sacrificed under anesthesia and the whole scapula including the implants was harvested for analysis.

### Radiographic evaluation

Radiographical analysis was performed at 4, 8, and 12 weeks postoperatively (DR3000 Dryview8900, Koda, Japan). The anteroposterior view of the scapula was obtained by X-ray. All radiographic results were evaluated under randomized and double-blind conditions. The Lane-Sandhu scoring system (Table 1) was applied to evaluate radiographic outcomes [21]. Radiographic scores were compared between the three groups.

**Table 1:** Lane–Sandhu radiographic scoring standard

Category	Standard	Scores
Bone formation	No evidence of bone formation	0
	Bone formation occupying 25% of defect	1
	Bone formation occupying 50% of defect	2
	Bone formation occupying 75% of defect	3
	Full gap bone formation	4
Fracture line	Clear	0
	Relatively clear	1
	Partial fracture line	2
	Basically vanished	3
	Completely vanished	4
Bone remodelling	No evidence of remodelling	0
	Remodelling of medullary canal	2
	Full remodeling of cortex	4

### Histological evaluation

Middle area tissue in experimental sites of the scapulae was excised out as specimens after X-ray radiographic examination. The specimens were fixed with 4% neutral-buffered formalin for 3 days and decalcified with 10% EDTA-2Na solution for 4 weeks at 4 °C. Then they were dehydrated with an ascending series of ethanol solutions, and cut into serial paraffin sections by the conventional method. Specimens were stained with hematoxylin and eosin (HE) and Masson’s trichrome for histological analysis.

All sections from each specimen were evaluated by light microscopy. Image analysis software (Pro-image Analysis System) was used to evaluate all sections from each specimen. The Lane–Sandhu scoring system (Table 2) was applied to evaluate histological outcomes [21]. Histological scores were compared between the three groups.

**Table 2:** Lane–Sandhu histological score standard

Category	Standard	Scores
Union	No sign of union	0
	Fibrous union	1
	Osteochondral union	2
	Bone union	3
	Complete reorganization	4
Spongiosa	No sign of cellular activity	0
	Early bone formation	1
	Active new bone formation	2
	Reorganized spongiosa formation	3
	Complete reorganized spongiosa	4
Cortex	Absence of cortex	0
	Early detection	1
	Initiation of formation	2
	Reorganization in majority	3
	Complete organization	4

### Ink-formaldehyde perfusion

A rabbit, randomly selected from four rabbits in Group 1 at 12 weeks, was introduced an ink-perfusion for discriminating vascularization of TEP mediated bone regeneration. The procedure was administered according to the method of Jian Y [22], but with a little bit of modification. Briefly, the rabbit was accepted a general anesthesia intraperitoneally with an injection of 3% pentobarbital solution (40 mg/kg body weight). Subsequently, whole body heparinization was accomplished by administering an intramuscular injection of 1000 U/kg heparin. The skin and subcutaneous tissues of axillary region was cut open to expose the axillary artery and vein. Then, the axillary artery was ligated severed and cannulated distally. The axillary vein was severed, then bled from the distal end. A large quantity of heparin-saline (heparin 12,500 U; saline 500 mL) was infused from the distal end of the axillary artery until the outflow from the axillary vein was clear. A kind of perfusate solution mixed with 30 vol% Chinese ink, 10 vol% formaldehyde and 60 vol% saline was injected into the axillary artery through the cannula until the outflow from the axillary vein was black and the skin and hooves turned black. The femoral axillary and vein then were ligated. The rabbits were sacrificed and the scapula was excised, following stored at 4 °C for 24 h, then, fixed with formaldehyde (40 g/L) for 1 week. The bone specimen was sliced transversely and longitudinally into 100–200 μm sections with a sawing microtome (Leica SP 1600; Leica Microsystems, Germany). Slides were stained with hematoxylin and eosin (HE) for observing microvessels formation in newly formed bone.

### Statistical analysis

Statistical analysis was performed using SPSS 15.0 software. All quantitative data are expressed as the mean ± standard deviation (M ± SD). Statistical comparison was performed by one-way analysis of variance (ANOVA). Statistical significance was considered at a probability < 0.05.

## Results

### Osteogenic differentiation of MSCs

**Fig. 2:**
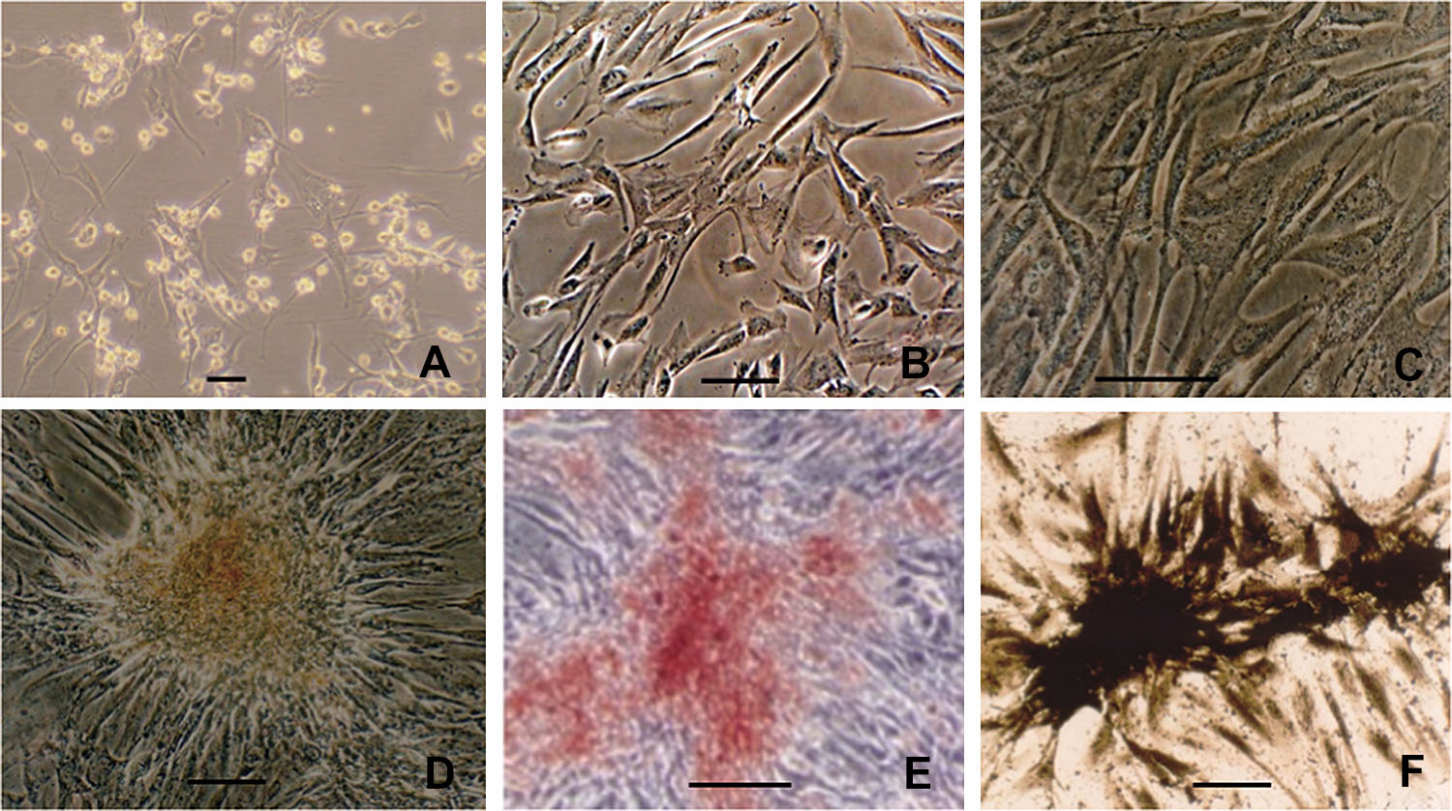
Seeding of cells. **A** MSCs of primary passage; **B** Uniform morphology of the 3rd passage MSCs; **C** confluent MSCs induced with osteogenic media showed intracellular refractive granules; **D** after 3-week induction, MSCs clused and formed calcified nodules; **E** calcified nodules formation from osteogenically-induced MSCs confirmed by alizarin red staining; **F** ALP expression of osteogenically differentiated MSCs visualized by the modified Gomori staining. Scale bar = 100 μm

Under a light microscope, the primary MSCs showed a triangular or polygonal appearance (Fig. 2A), then became uniform elongated fibroblast-like morphology at passage 3 (Fig. 2B). After osteogencally induced for 3 weeks in over confluent, there were refractive intracellular granules under light microscopy (Fig. 2C). Cells were inclined to cluster and form calcified nodules (Fig. 2D), which were further confirmed by Alizarin Red staining (Fig. 2E). The modified Gomori staining showed intensive ALP expression in osteogencally induced MSCs (Fig. 2F). These signs indicated that the induced MSCs osteogenically differentiated.

### Characteristics of SIS and SEM

**Fig. 3:**
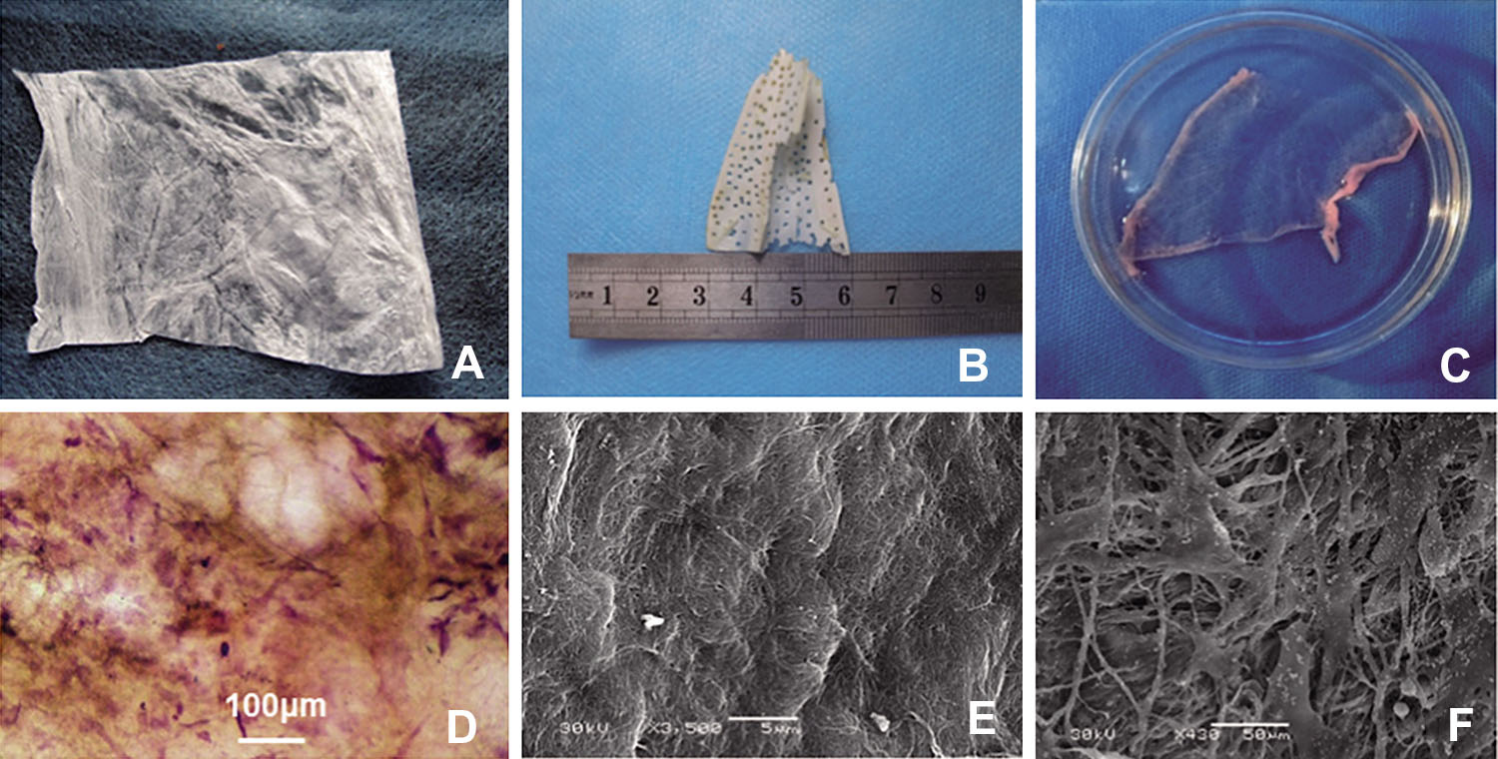
Implants of bone defects. **A** Macroscopical appearance of SIS; **B** macroscopical appearance of DPB derived of scapula block; **C** macroscopical appearance of TEP in culture; **D** seeding cells attached on TEP under light microscope, visualized by HE staining (scale bar = 100 μm); **E** SIS visualized by SEM; **F** seeding cells attached on TEP, visualized by SEM

When examined macroscopically, the homemade SIS scaffold appeared as a white and flexible membrane with a thickness of 100 ± 20 μm (Fig. 3A). The DPB implants were shaped into scalene triangles 5 ± 2 mm in thickness, with multiple holes (Fig. 3B). The TEP implants preserved the flexible nature and showed a membranous structure in gross view in a culture dish (Fig. 3C). HE staining showed multiple cells on the TEP (Fig. 3D). Under SEM, SIS consisted of interlaced collagen fibers (Fig. 3E), while plenty of cells could be seen attached to the TEP on SIS (Fig. 3F).

### Animal behaviors of postoperation

The animals rapidly recovered within 1 h after surgery and could stand well in 24 h. All wounds closed within 1 week without any observable infection of the incision. In approximately 2 weeks, they were able to move freely. All animals remained in normal health throughout the course of the experiment. There was no evidence of infection or other complications in any animal.

### Radiographic outcome

The progress of bone defect repair was analyzed by radiography with a parallel comparison between Groups 1, 2, and 3 at 4, 8, and 12 weeks.

**Fig. 4:**
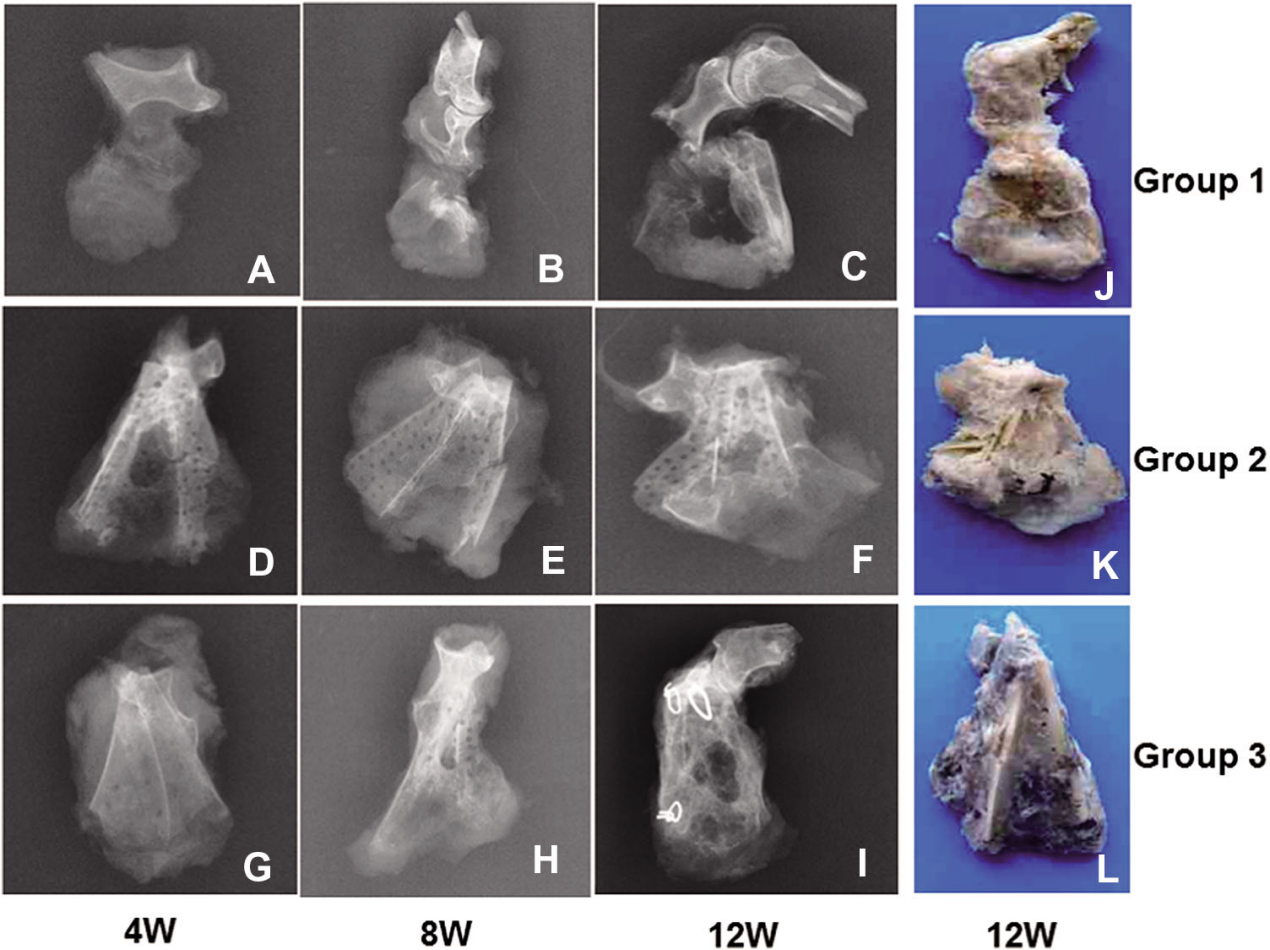
X-ray radiographic and macroscopic observation. Top row (**A**–**C**) represents X-ray radiographs of scapular defect repair in Group 1 at 4, 8 and 12 weeks respectively. The 2nd row (**D**–**F**) represents X-ray radiographs of scapular defect repair in Group 2 at 4, 8 and 12 weeks respectively. The 3rd row (**A**–**C**) represents X-ray radiographs of scapular defect repair in Group 3 at 4, 8 and 12 weeks respectively. The 4th column (**J**–**L**) represents macroscopic views of scapular defect repair in Group 1, 2, and 3, respectively, at 12 weeks

In Group 1, low density calluses were observed in the bone defect area at 4 weeks postoperation (Fig. 4A). At 8 weeks, more calluses were observed (Fig. 4B). At 12 weeks, the volume of newly-formed bone and the bone mineral density were both greatly increased (Fig. 4C). Bony union was achieved according to the bone healing criteria reported in the literature [9].

In Group 2, there was very little DPB absorption and little callus formation observed at 4 weeks postoperation (Fig. 4D). At 8 weeks, DPB absorption was observed with the appearance of a small amount of callus in the bone defect area (Fig. 4E). At 12 weeks, the DPB was absorbed further with some new bone formation (Fig. 4F).

In Group 3, the holes in the DPB could not be clearly seen due to the growth of new callus within them by 4 weeks (Fig. 4G). At 8 weeks, there was much more newly formed bone in the bone defect area. The DPB was partially degraded and new bone formation was observed under X ray inspection. The bone mineral density was also greatly increased (Fig. 4H). At 12 weeks postoperation, newly-formed bone was substituted for degraded grafts (Fig. 4I).

**Fig. 5:**
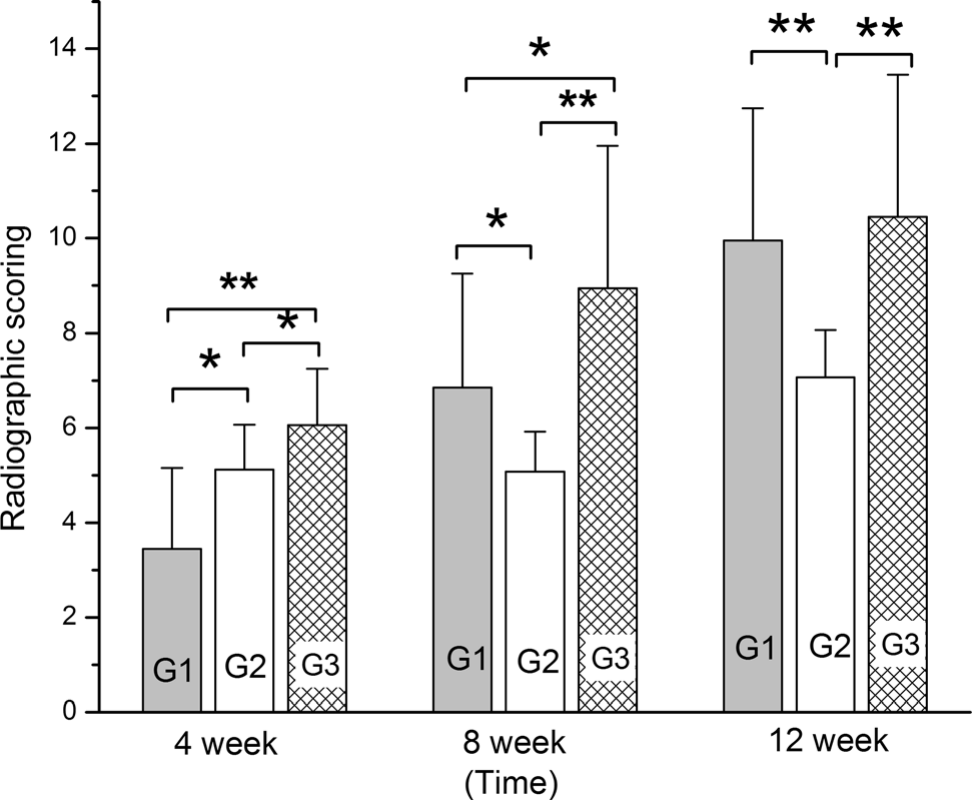
Histogram of radiographic scores between each groups at 4, 8, and 12 weeks. “*” and “**” represents a significant difference between different groups at the same time (*p < 0.05 and **p < 0.001). G1, 2 and 3 respectively represents Groups 1, 2, and 3

As the intergroup comparison was concerned, the radiographic score in Group 3 was significantly higher than in Group 1 (*p* < 0.001, at 4 weeks) or Group 2 at 4 and 8 weeks (*p* < 0.05). The score in Group 1 was significantly lower than Group 2 at 4 weeks (*p* < 0.05), while it increased significantly higher than that in Group 2 at 8 weeks (*p* < 0.05) and 12 weeks (*p* < 0.001). Meanwhile, the score in Group 1 drew level with that in Group 3 at 12 weeks (*p* > 0.05) (Fig. 5).

### Histological findings

The samples excised from the scapular area were very similar to each other when examined macroscopically, and formed the approximate shape of a scapula at 12 weeks (Fig. 4J, K, and L).

Under the microscope, modest monocyte infiltration was evident at 4 weeks in each group, visualized by HE staining.

**Fig. 6:**
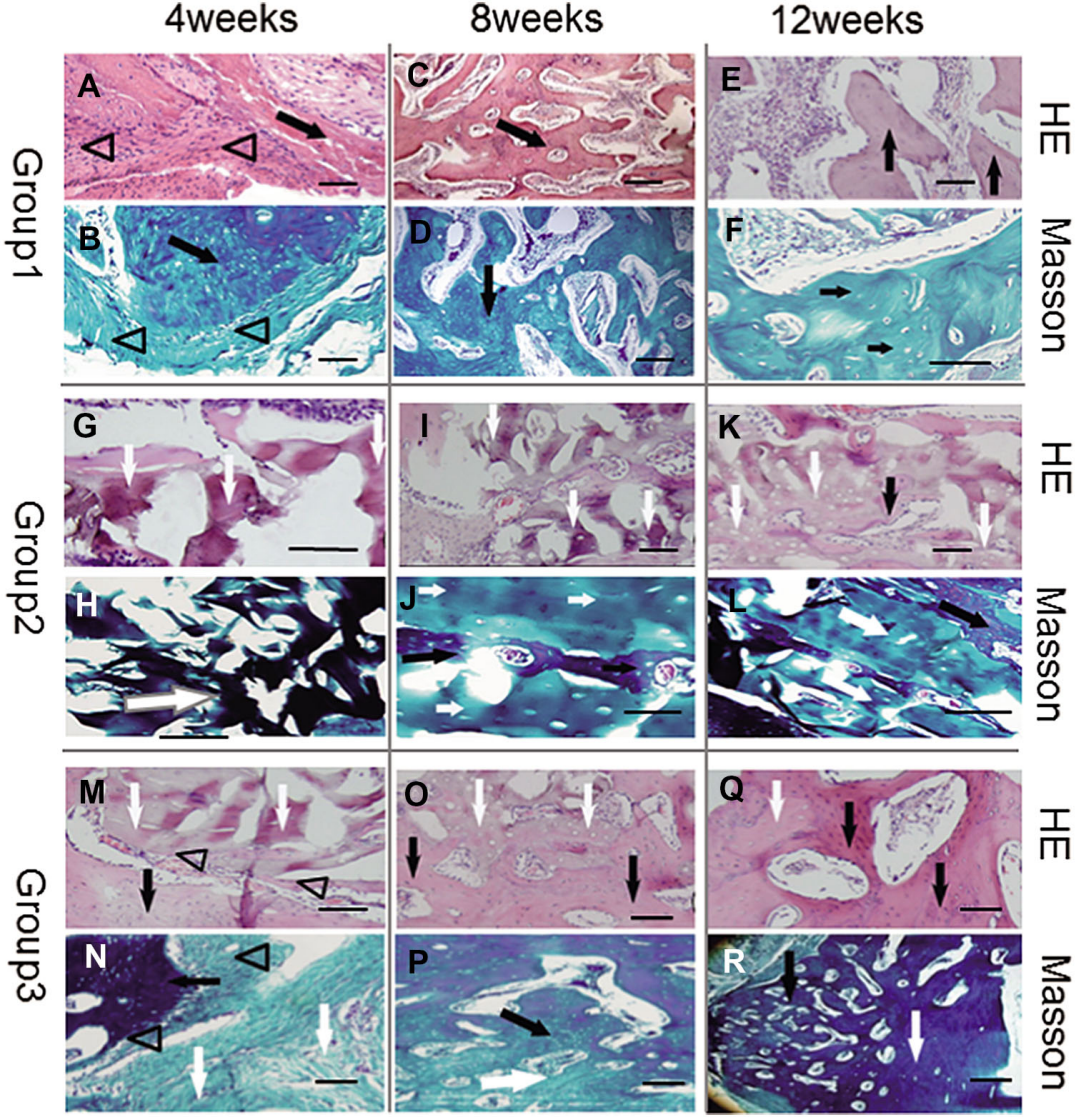
Histological examination under light microscope with HE (row 1, 3 and 5) and Masson’s trichrome (row 2, 4, and 6) staining. In Group 1 (row 1, 2), TEP formed island-like calluses at 4 weeks (**A**, **B**). New bone tissue increased with irregular vessels or immature marrow cavities, while TEP disappeared (possibly degraded) at 8 weeks (**C**, **D**). At 12 weeks, all newly-formed bone developed into mature cancellous bone (**E**, **F**). In Group 2 (rows 3, 4), DPB was mainly surrounded by scar tissue and infiltrated lymphocytes at 4 weeks (**G**, **H**), and accompanied by little new osseous tissue formation at 8 (**I**, **J**) or 12 weeks (**K**, **L**). In Group 3 (rows 5, 6), there was a small amount of new bone formation between TEP and DPB, which were degraded accompanied by scar tissue at 4 weeks (**M**, **N**). New osseous tissue formed woven bone at 8 weeks (**O**, **P**), while it was inclined to form mature lamellar bone with osteoblasts embedded into mineral matrix at 12 weeks (**Q**, **R**). Triangles represent TEP, black arrows represent newly-formed bone tissue, white arrows represent remnants of DPB. Scale bar = 1 mm

In Group 1, newly-formed immature osseous tissue and irregular vessels could be seen in the midst of degraded residual SIS and fibrous connective tissue. At 8 weeks, new osseous tissue was partially formed into woven bone with mature vessels, while the TEP was almost degraded with few fibrous remnants and much scar tissue. By 12 weeks, all newly-formed bone had developed into mature cancellous bone under HE and Masson staining inspection (Fig. 6A–F).

In Group 2, DPB was degraded gradually at 4 and 8 weeks accompanied with a small amount of new osseous tissue formation. The new bone tissue was significantly more abundant at 12 weeks than at 4 or 8 weeks (*p* < 0.05). However the bone defect area was predominantly occupied by DPB even at 12 weeks (Fig. 6G–L).

In Group 3, areas of new osseous tissue formation were accompanied by SIS and DPB degradation at 4 weeks. New osseous tissue formed woven bone at 8 weeks, while it was inclined to form mature lamellar bone with osteoblasts embedded into mineral matrix at 12 weeks. Remnants of DPB were still abundant in the defect area even at 12 weeks (Fig. 6M–R).

**Fig. 7:**
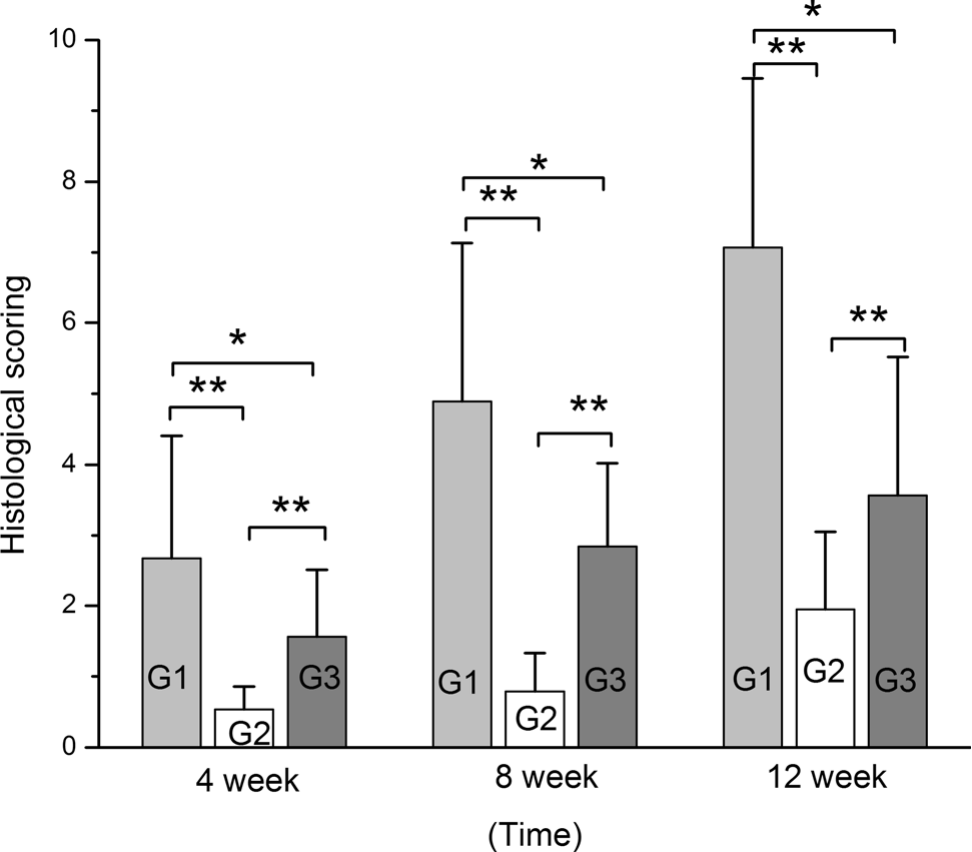
Histogram of histological scores of each group at 4, 8, and 12 weeks. “*” and “**”represent significant differences between the different groups at the same time (*p < 0.05 and **p < 0.001). G1, 2, and 3 respectively represent Groups 1, 2, and 3

In comparison, Group 1 appeared to possess higher bone formation than Group 2 or 3 at 4, 8, and 12 weeks (*p* < 0.05 between Group 3, *p* < 0.001 between Group 2). Group 3 also exhibited higher new bone volume than that of Group 2 at 4, 8, and 12 weeks (*p* < 0.001) (Fig. 7).

### Vascularizaiton of TEP mediated bone regeneration

**Fig. 8:**
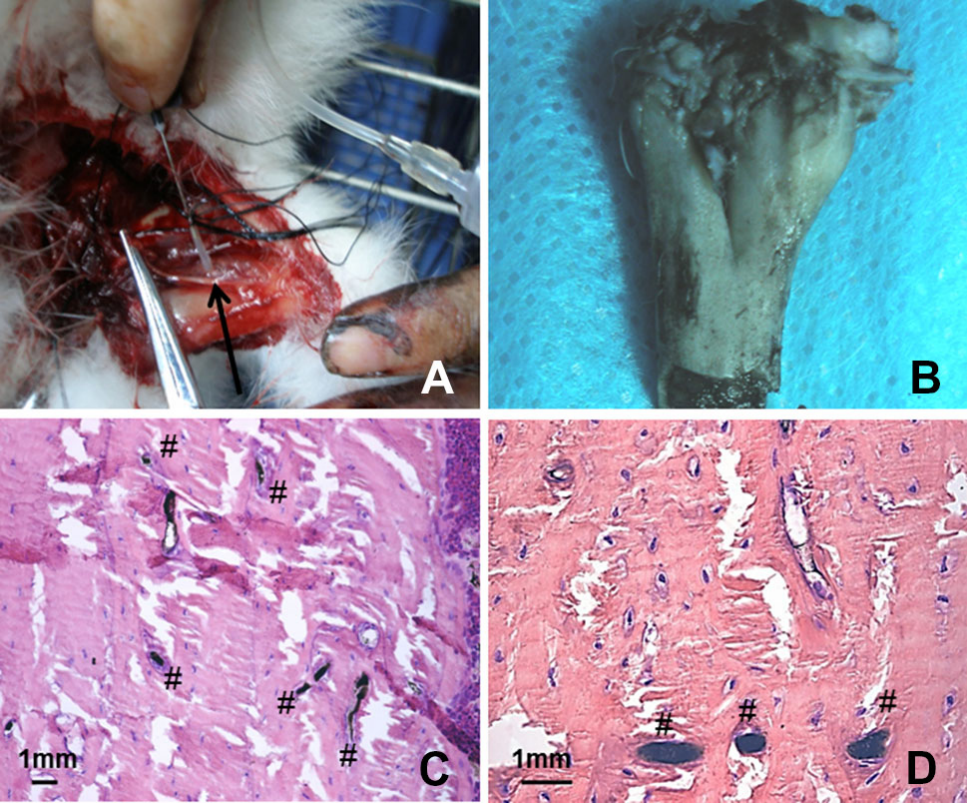
Ink-formaldehyde perfusion. **A** Surgery of perfusion through axillary artery; black arrow points to axillary artery; **B** macroscopical appearance of the bone block after ink-perfusion. **C** black microvessels filled with black ink visible as stripes in the longitudinal sections, and round dots in the transverse sections (**D**). “#” represents ink filled microvessel; Scale bar = 1 mm

After ink-formaldehyde perfusion through axillary artery (Fig. 8A), whole bone block was blackened. Macroscopically, there were some small vessels and reticular anastomosis observed on the surface of the bone block (Fig. 8B). The specimen of newly formed bone from Group 1(TEP implanted group) was sliced transversely and longitudinally sections to observe microvessels with HE staining. Under light microscope, osteocytes, osseous tissue, marrow cavity were clearly discernible, and black microvessels filled with black ink were clearly visible as stripes in the longitudinal sections (Fig. 8C) and round dots (Fig. 8D) in the transverse sections.

## Discussion

In the present study as well as our previous experiments, we fabricated a 2D periosteum-equivalent structure based on tissue-engineering principles, which we named TEP. We propose that this kind of flexible cellular loading construct may mimic a periosteal response during initiation of bone defect repair.

The TEP is fabricated by combining SIS with osteoblasts (induced from MSCs), and offers advantages over other tissues as a graft material, because it is easy to handle and enhances vascularization [17, 18, 23]. Moreover, it produces a minimal immune reaction [24]. The thickness (100 ± 20 μm) of SIS is lower than the critical value (500 μm), and thus can permit attached cells to survive on nutrients diffused from the interstitial fluid in the early stage after implantation [25]. Successful bone formation was observed in both Groups 1 and 3 using this TEP approach in this study. The findings suggested that TEP might regenerate bone tissue directly via the surviving cells attached to it. This suggested that any size and shape of bone defect could be successfully repaired using this simulated periosteal approach without pre-vascularization of constructs, which has been a critical challenge in the area of tissue engineering until now [26, 27].

Histological inspection showed that the volume of newly-formed bone in Group 1 was significantly greater than that in Group 2 or 3, and that in Group 3 was significantly higher than in Group 2 in the same period, which indicated that TEP had osteogenic bioactivity. These findings were in accordance with the results of our previous studies in rabbit models of long bone defects [17, 18].

In this study, the radiographic score in rabbits receiving TEP-DPB hybrid implantation (Group 3) was higher than those receiving TEP or DPB grafting alone. However this was inconsistent with histological findings. Decellularization protocols have to efficiently remove immunogenic materials, while retaining the nonimmunogenic ECM, which is endowed with specific inductive activity due to its architecture and bioactive factors [28]. As control scaffold in this study in Groups 2 and 3, we used DPB from allogeneic scapulae, which retains the 3D structure, porous microstructure and osteoinductive nature and provides a relatively safe bone graft material since it is free of proteins that might induce immune rejection [29]. Meanwhile, DPB was degraded in vivo and substituted by new bone, but this osseointegration is a slow process called creeping substitution [14, 30]. It might be the reason that new bone formation in Group 2 was slower than in Group 1 or 3, and the radiological result was inconsistent with histological findings.

In Group 3, implanted with DPB combined with TEP, the former served as a shape-guiding scaffold and mechanical support for TEP during new bone regeneration, while the latter constituted a vital component endowed with osteogenic bioactivity. This hybrid was expected to induce abundant new bone formation due to the biomimetic periosteum and in accordance with the shape of the DPB scaffold. The findings showed that there was more newly-formed bone tissue in Group 3 than Group 2, but less than Group 1, although the approximate shape of the DPB scaffold was maintained. We speculate that this might be due to resorption of the DPB scaffold being slower than new bone formation via the TEP approach.

In Group 1, new bone formation was abundant, and formed the approximate shape of the scapular body, although there was no shape-guiding 3D scaffold. The tensioned TEP attached to three anchor points, as described above in the surgical section, maintained the triangular shape in Group 1, and that might provide the contour of irregular bone formation in a shape approximating the scapular body. Moreover, regeneration of new bone was more abundant than in Group 3, in which bone formation might be hindered by delayed degradation of the DPB scaffold. As reported in the literature, remodeling of allograft bone occurs at a very slow rate and necrotic bone cannot be completely replaced by new bone [31, 32]. This means that in BTE, a balance must be struck between scaffold absorption and new bone formation which will be important in future study and will improve practicality for widespread clinical use [23, 33].

Vascularity is essential for providing the optimal blood supply to maintain survival of the osteogenic cells [34, 35]. In this study, the 2D structure of TEP could permit attached cells to survive on diffused nutrients from the interstitial fluid in the early stage [28]. Moreover, SIS used as the scaffold of TEP in this study has strong angiogenic effects and retains plentiful bioactive components such as vascular endothelial growth factor (VEGF) [19, 36]. This advantage of SIS might contribute partially to vascularization during the latter stage of bone regeneration. In the present study, TEP in vitro fabrication was simplified with the use of only two components, SIS and MSCs, and we just used the body as an in vivo bioreactor to regenerate new bone as well as accompanying vessels, nerves and other affiliated tissues, according to the tissue engineering concept of some researchers [5, 33]. In this study, we used ink- formaldehyde perfusion and HE staining to visualize vasculariztion of newly engineered bone regenerated from TEP. Small vessels and reticular anastomosis were seen under macroscopy. The microvessels were clearly discerned under light microscopy, though the area, number, size and direction of microvessels were diffcult to assess with this method [22]. Therefore, in vivo osteogenesis and angiogenesis might be much more complicated beyond our present speculation, and will need intensive study in future.

The components of TEP: MSCs and SIS, have great immune advantages and are suitable for allograft [24]. This therefore provides the promise of off-the-shelf products for future clinical application. Tissue-engineered bone generated via the TEP approach in this study demonstrated formation of the approximate shape of the scapula. In future, customized fabrication techniques, such as three-dimensional printing, with intelligent materials, whose degradation would be synchronized with new bone formation, would provide a precise repair for segmentally irregular bone defects via the TEP approach [1, 4].

In this study, there are several limitations that will need further investigation. First, we have not tested mechanical properties and degradation profiles of TEP and hybrid of TEP/DPB. Second, seeding efficiency or cell density on TEP has not been calculated, which may impact standardization of TEP fabrication. Third, besides of SIS implanted group (negative control), it may be necessary to design a sham operated group of no treatment in bone defects to more clearly confirm in vivo osteogenic potential of TEP.

## References

[1] Zheng X, Huang J, Lin J, Yang D, Xu T, Chen D (2019). 3D bioprinting in orthopedics translational research. J Biomater Sci Polym Ed.

[2] Jiang H, Cheng P, Li D, Li J, Wang J, Gao Y (2018). Novel standardized massive bone defect model in rats employing an internal eight-hole stainless steel plate for bone tissue engineering. J Tissue Eng Regen Med..

[3] Shanbhag S, Pandis N, Mustafa K, Nyengaard JR, Stavropoulos A (2017). Cell cotransplantation strategies for vascularized craniofacial bone tissue engineering: a systematic review and meta-analysis of preclinical in vivo studies. Tissue Eng Part B Rev.

[4] Huang YH, Jakus AE, Jordan SW, Dumanian Z, Parker K, Zhao L (2019). Three-dimensionally printed hyperelastic bone scaffolds accelerate bone regeneration in critical-size calvarial bone defects. Plast Reconstr Surg.

[5] Huang RL, Liu K, Li Q (2016). Bone regeneration following the in vivo bioreactor principle: is in vitro manipulation of exogenous elements still needed?. Regen Med.

[6] Landman KA, Cai AQ (2007). Cell proliferation and oxygen diffusion in a vascularising scaffold. Bull Math Biol.

[7] Santos MI, Reis RL (2010). Vascularization in bone tissue engineering: physiology, current strategies, major hurdles and future challenges. Macromol Biosci.

[8] Yu H, VandeVord PJ, Mao L, Matthew HW, Wooley PH, Yang SY (2009). Improved tissue-engineered bone regeneration by endothelial cell mediated vascularization. Biomaterials.

[9] Lin CY, Chang YH, Lin KJ, Yen TC, Tai CL, Chen CY (2010). The healing of critical-sized femoral segmental bone defects in rabbits using baculovirus-engineered mesenchymal stem cells. Biomaterials.

[10] Fidkowski C, Kaazempur-Mofrad MR, Borenstein J, Vacanti JP, Langer R, Wang Y (2005). Endothelialized microvasculature based on a biodegradable elastomer. Tissue Eng.

[11] Percival CJ, Richtsmeier JT (2013). Angiogenesis and intramembranous osteogenesis. Dev Dyn.

[12] Orwoll ES (2003). Toward an expanded understanding of the role of the periosteum in skeletal health. J Bone Miner Res.

[13] Wlodarski KH (1989). Normal and heterotopic periosteum. Clin Orthop Relat Res.

[14] Zhang X, Awad HA, O’Keefe RJ, Guldberg RE, Schwarz EM (2008). A perspective: engineering periosteum for structural bone graft healing. Clin Orthop Relat Res.

[15] Ouyang HW, Cao T, Zou XH, Heng BC, Wang LL, Song XH (2006). Mesenchymal stem cell sheets revitalize nonviable dense grafts: implications for repair of large-bone and tendon defects. Transplantation.

[16] Knothe Tate ML, Ritzman TF, Schneider E, Knothe UR (2007). Testing of a new one-stage one-transport surgical procedure exploiting the periosteum for the repair of long-bone defects. J Bone Joint Surg Am.

[17] Zhao L, Zhao J, Yu J, Sun R, Zhang X, Hu S (2017). In vivo investigation of tissue-engineered periosteum for the repair of allogeneic critical size bone defects in rabbits. Regen Med..

[18] Zhao L, Zhao J, Yu J, Zhao X, Chen Q, Huang Y (2016). In vitro study of bioactivity of homemade tissue-engineered periosteum. Mater Sci Eng C Mater Biol Appl.

[19] Luo JC, Chen W, Chen XH, Qin TW, Huang YC, Xie HQ (2011). A multi-step method for preparation of porcine small intestinal submucosa (SIS). Biomaterials.

[20] Zhao M, Zhou J, Li X, Fang T, Dai W, Yin W (2011). Repair of bone defect with vascularized tissue engineered bone graft seeded with mesenchymal stem cells in rabbits. Microsurgery.

[21] Zhang L, Mu W, Chen S, Yang D, Xu F, Wu Y (2016). The enhancement of osteogenic capacity in a synthetic BMP-2 derived peptide coated mineralized collagen composite in the treatment of the mandibular defects. Biomed Mater Eng.

[22] Jian Y, Tian X, Li Q, Li B, Peng Z (2012). Comparison of methods for staining microvessels in bone. Biotech Histochem.

[23] Cancedda R, Giannoni P, Mastrogiacomo M (2007). A tissue engineering approach to bone repair in large animal models and in clinical practice. Biomaterials.

[24] Zhao L, Zhao J, Wang S, Xia Y, Liu J, He J (2011). Evaluation of immunocompatibility of tissue-engineered periosteum. Biomed Mater.

[25] Ye H, Xia Z, Ferguson DJ, Triffitt JT, Cui Z (2007). Studies on the use of hollow fibre membrane bioreactors for tissue generation by using rat bone marrow fibroblastic cells and a composite scaffold. J Mater Sci Mater Med.

[26] Sasaki JI, Katata C, Abe GL, Matsumoto T, Imazato S (2019). Fabricating large-scale three-dimensional constructs with living cells by processing with syringe needles. J Biomed Mater Res A.

[27] Mi HY, Jiang Y, Jing X, Enriquez E, Li H, Li Q (2019). Fabrication of triple-layered vascular grafts composed of silk fibers, polyacrylamide hydrogel, and polyurethane nanofibers with biomimetic mechanical properties. Mater Sci Eng C Mater Biol Appl.

[28] Porzionato A, Stocco E, Barbon S, Grandi F, Macchi V, De Caro R (2018). Tissue-engineered grafts from human decellularized extracellular matrices: a systematic review and future perspectives. Int J Mol Sci.

[29] Zhou Y, Guan X, Wang H, Zhu Z, Li C, Wu S (2013). Hypoxia induces osteogenic/angiogenic responses of bone marrow-derived mesenchymal stromal cells seeded on bone-derived scaffolds via ERK1/2 and p38 pathways. Biotechnol Bioeng.

[30] Cobos JA, Lindsey RW, Gugala Z (2000). The cylindrical titanium mesh cage for treatment of a long bone segmental defect: description of a new technique and report of two cases. J Orthop Trauma.

[31] Burchardt H (1987). Biology of bone transplantation. Orthop Clin North Am.

[32] Bauer TW, Muschler GF (2000). Bone graft materials. An overview of the basic science. Clin Orthop Relat Res.

[33] Service RF (2005). Tissue engineering. Technique uses body as ‘bioreactor’ to grow new bone. Science.

[34] Leunig M, Demhartner TJ, Sckell A, Fraitzl CR, Gries N, Schenk RK (1999). Quantitative assessment of angiogenesis and osteogenesis after transplantation of bone: comparison of isograft and allograft bone in mice. Acta Orthop Scand.

[35] Leunig M, Yuan F, Berk DA, Gerweck LE, Jain RK (1994). Angiogenesis and growth of isografted bone: quantitative in vivo assay in nude mice. Lab Invest.

[36] Sun T, Liu M, Yao S, Ji Y, Xiong Z, Tang K (2018). Biomimetic composite scaffold containing small intestinal submucosa and mesoporous bioactive glass exhibits high osteogenic and angiogenic capacity. Tissue Eng Part A.

